# Supplementary material for: Atlas of amnion development during the first trimester of human pregnancy
Source: Nat Cell Biol. 2025 Jul 14;27(7):1175–85. doi: 10.1038/s41556-025-01696-9 (PMC12270915; doi:10.1038/s41556-025-01696-9)
Supplement: Supplementary file 1 — Reporting Summary [file 41556_2025_1696_MOESM1_ESM.pdf]

nature portfolio

Corresponding author(s): Magdalena Zernicka-Goetz

Last updated by author(s): Apr 29, 2025

Reporting Summary

Nature Portfolio wishes to improve the reproducibility of the work that we publish. This form provides structure for consistency and transparency in reporting. For further information on Nature Portfolio policies, see our Editorial Policies and the Editorial Policy Checklist.

Statistics

For all statistical analyses, confirm that the following items are present in the figure legend, table legend, main text, or Methods section.

n/a	Confirmed	
☐	☒	The exact sample size (*n*) for each experimental group/condition, given as a discrete number and unit of measurement
☐	☒	A statement on whether measurements were taken from distinct samples or whether the same sample was measured repeatedly
☐	☒	The statistical test(s) used AND whether they are one- or two-sided<br>*Only common tests should be described solely by name; describe more complex techniques in the Methods section.*
☒	☐	A description of all covariates tested
☐	☒	A description of any assumptions or corrections, such as tests of normality and adjustment for multiple comparisons
☐	☒	A full description of the statistical parameters including central tendency (e.g. means) or other basic estimates (e.g. regression coefficient) AND variation (e.g. standard deviation) or associated estimates of uncertainty (e.g. confidence intervals)
☐	☒	For null hypothesis testing, the test statistic (e.g. *F*, *t*, *r*) with confidence intervals, effect sizes, degrees of freedom and *P* value noted<br>*Give P values as exact values whenever suitable.*
☒	☐	For Bayesian analysis, information on the choice of priors and Markov chain Monte Carlo settings
☒	☐	For hierarchical and complex designs, identification of the appropriate level for tests and full reporting of outcomes
☒	☐	Estimates of effect sizes (e.g. Cohen's *d*, Pearson's *r*), indicating how they were calculated

Our web collection on statistics for biologists contains articles on many of the points above.

Software and code

Policy information about availability of computer code

Data collection	For high-throughput sequencing data collection, we used lllumina HiSeq4000 or Nov aseq 6000 systems
Data analysis	For high throughput sequencing data processing and subsequent data analyses we used:<br>linux packages: Cell Ranger(8.0.l), Souporcell(v2.5)<br>R packages: R(4.2.2), Seurat(4.3.0), monocle3 (1.3.l),dplyr(l.l.2), CellChat(l.6.1), destiny(3.20)<br>python packages: Python(3.ll.0), ScVelo(0.3.2), ScanPy(l.10.1), pandas(2.2.2), anndata(0.10.7)<br>Tools: Graph Prism(8.2.0), Metascape(v3.5)

For manuscripts utilizing custom algorithms or software that are central to the research but not yet described in published literature, software must be made available to editors and reviewers. We strongly encourage code deposition in a community repository (e.g. GitHub). See the Nature Portfolio guidelines for submitting code & software for further information.

Data

Policy information about availability of data

All manuscripts must include a data availability statement. This statement should provide the following information, where applicable:

- Accession codes, unique identifiers, or web links for publicly available datasets
- A description of any restrictions on data availability
- For clinical datasets or third party data, please ensure that the statement adheres to our policy

Sequencing data that support the findings of this study have been deposited in the Gene Expression Omnibus (GEO) under accession code GSE260715. Previously published human CS7 embryo data was downloaded from Array Express: E-MTAB-9388. Monkey CS8_CS11 data was obtained from GEO under accession number GSE193007. Stem cell derived model data were downloaded from GSE179309, GSE205611 and GSE134571. The human reference genome (GRCh38/hg38) used for alignment was downloaded from the 10x Genomics website (https://cf.10xgenomics.com/supp/cell-exp/refdata-gex-GRCh38-2020-A.tar.gz). Source data are provided with this study. All other data supporting the findings of this study are available from the corresponding author on reasonable request.

Research involving human participants, their data, or biological material

Policy information about studies with human participants or human data. See also policy information about sex, gender (identity/presentation), and sexual orientation and race, ethnicity and racism.

Reporting on sex and gender	We collected amnion tissue from embryos in the first trimester. Sex and genders are not identified in this study.
Reporting on race, ethnicity, or other socially relevant groupings	Race, ethnicity, or other socially relevant groupings are not identified in this study.
Population characteristics	We collected four human amnion samples representing weeks 5, 6, 7 and 8 of pregnancy and corresponding to Carnegie stages (CS) 16, 17, 19, and 22, respectively. Detailed covariate information such as age, genotype, or medical history of the donors was not available. All participants were healthy pregnant women who provided informed consent for the donation of amnion tissue.
Recruitment	Human amnion tissue samples were obtained through the MRC-Wellcome Trust Human Developmental Biology Resource (HDBR), from elective terminations of pregnancy with informed consent. Donors were not actively recruited by the research team. As samples were only obtained from individuals who consented to tissue donation for research purposes, potential self-selection bias may exist. Additionally, all samples were collected at collaborating clinical centers in the UK, which may limit generalizability to other populations. However, no information was available regarding the donors' demographic or clinical background beyond confirmation of healthy pregnancy status.
Ethics oversight	All procedures were approved by the MRC-Wellcome Trust Human Developmental Biology Resource (HDBR) under ethical approval from the London - Fulham Research Ethics Committee (Reference: 08/H0712/34+5, IRAS Project ID: 134561). Sample collection followed HDBR standard operating procedures and documentation, including: Patient Information Sheet and Consent Form, version 16; SOP – Recruitment of Donors, version 8; SOP – Collection of Consented Material, version 7; HDBR Background and Protocol, version 10.

Note that full information on the approval of the study protocol must also be provided in the manuscript.

Field-specific reporting

Please select the one below that is the best fit for your research. If you are not sure, read the appropriate sections before making your selection.

☒ Life sciences ☐ Behavioural & social sciences ☐ Ecological, evolutionary & environmental sciences

For a reference copy of the document with all sections, see nature.com/documents/nr-reporting-summary-flat.pdf

Life sciences study design

All studies must disclose on these points even when the disclosure is negative.

Sample size	No statistical methods were used to pre-determine sample size. Human amnion tissues were limited in availability, and all suitable samples from consenting donors were included in the study. Sample sizes are comparable to those reported in previous human single-cell studies. Across all samples, over 14,000 single cells were profiled, which provided sufficient resolution to identify cell populations and key gene expression patterns relevant to early development.
Data exclusions	To exclude potential maternal contamination, in our single-cell analysis, we carried out souporcell during data analysis. Cells from a single genotype that cluster into a Seurat cluster are identified as maternal cells and are excluded from the analysis.<br>In addition, we used the "AddModuleScore" function in Seurat package to assign identify (score) and exclude: erythrocytes (markers: HBZ, HBEl, HBG2, HBGl, HBAl, HBA2, HBM, ALAS2, HBB, GYPB, GYPC, GYPA), chorion (markers: CGA, CGB3, GCMl, CGBS, CGB7, CGB8), yolk sac

	(markers: AFP, CERl, HHEX, FOXA2, SPIN Kl), and blood vessels (markers: CD34, PECAMl, CLDNS, CDHS, ESAM, FLTl, OGN). Only cells with scores <0 were kept for analysis
Replication	Replication was not applicable to single-cell RNA-seq samples as tissue availability from human donors was limited. All suitable samples obtained were included in the analysis, and scRNA-seq provides cellular-level resolution that captures biological variability across thousands of individual cells, partially mitigating the need for technical replication.<br>Immunofluorescence staining experiments were independently performed for multiple markers across different donor samples. Representative images are shown. 4 repeats were performed for CD45/VIM co-staining.2 repeats were performed for CD45/E-Cadherin co-staining. 1 repeat was performed for VIM/E-Cadherin and N-Cadherin/KRT18 staining. 2 repeats were performed for GABRP/SOX2 staining. 4 repeats were performed for E-Cadherin/TUBB3 staining. 3 repeats were performed for SPP1/CD44/MIF staining.<br>Stem cell experiments were independently repeated 2 times with consistent results.
Randomization	scRNA sequencing samples were not allocated into experimental groups. All available tissue samples from consenting donors were processed and analyzed as a single cohort. As this study is observational and exploratory in nature, group allocation and covariate control are not applicable.<br>Immunofluorescence (IF) staining was performed on available tissue samples from different human donors. Samples were not assigned to experimental groups, and no randomization or blinding was applied.<br>For stem cell experiments, samples were assigned to control and treatment groups based on experimental design. Allocation was randomized
Blinding	The investigators were not blinded to sample identity during data collection or analysis, as the study was exploratory in nature and did not involve treatment allocation or intervention groups. The investigators were not blinded to group allocation during the stem cell experiments or data analysis.

Reporting for specific materials, systems and methods

We require information from authors about some types of materials, experimental systems and methods used in many studies. Here, indicate whether each material, system or method listed is relevant to your study. If you are not sure if a list item applies to your research, read the appropriate section before selecting a response.

Materials & experimental systems

n/a	Involved in the study	
☐	☒	Antibodies
☐	☒	Eukaryotic cell lines
☒	☐	Palaeontology and archaeology
☒	☐	Animals and other organisms
☒	☐	Clinical data
☒	☐	Dual use research of concern
☒	☐	Plants

Methods

n/a	Involved in the study	
☒	☐	ChIP-seq
☒	☐	Flow cytometry
☒	☐	MRI-based neuroimaging

Antibodies

Antibodies used	GABRP Polyclonal Antibody, Thermo Fisher, Cat#PA5-46830<br>Vimentin (5G3F10), cell signal technology, mAb #3390<br>SOX2 Monoclonal Antibody (Btjce), Thermo Fisher,Catalog # 14-9811-82, clone: Btjce<br>Osteopontin Monoclonal Antibody (2F10), Thermo Fisher, Cat# 14-9096-82, clone: 2F10<br>MIF Polyclonal Antibody, Thermo Fisher, Cat# PA5-27343<br>CD44 Monoclonal Antibody (IM7) Thermo Fisher, Cat# 14-0441-82, clone: IM7<br>CD324 (E-Cadherin) Monoclonal Antibody (DECMA-1),Thermo Fisher,Catalog # 16-3249-82,RRID:AB_10734213, clone: DECMA-1<br>CD45 (Intracellular Domain) (D9M8I) , Cell signal technology, Cat#13917T, clone: D9M8I<br>Anti-N Cadherin antibody [5D5], Abcam, Cat#ab98952, RRID:AB_10696943, clone: 5D5<br>Anti-Keratin 18 antibody, Sigmaaldrich, Cat#SAB4501665, RRID:AB_10746153<br>Purified anti-Tubulin β 3 (TUBB3) Antibody, Biolegend, Cat#801213, RRID:AB_2313773, clone: TUJ1<br>Donkey anti-Rabbit IgG (H+L) Highly Cross-Adsorbed Secondary Antibody, Alexa Fluor™ 488 Thermo Fisher Cat# A-21206<br>Donkey anti-Mouse IgG (H+L) Highly Cross-Adsorbed Secondary Antibody, Alexa Fluor™ 568 Thermo Fisher Cat# A10037<br>Donkey anti-Rat IgG (H+L) Highly Cross-Adsorbed Secondary Antibody, Alexa Fluor™ Plus 647 Thermo Fisher Cat# A48272<br>Goat anti-Rat IgG (H+L) Cross-Adsorbed Secondary Antibody, Alexa Fluor™ 647 Thermo Fisher Cat# A-21247
Validation	Anti-GABRP (Thermo Fisher, Cat# PA5-46830) is validated by the manufacturer for human samples in IHC and WB applications.<br>Anti-Vimentin (clone 5G3F10, Cell Signaling Technology, Cat# 3390) is a mouse monoclonal antibody validated by the manufacturer for immunofluorescence and human samples. The antibody has been widely used in published studies involving human tissues and cell lines, and produces a clear cytoplasmic staining pattern consistent with known Vimentin expression.<br>Anti-SOX2 (clone Btjce, Thermo Fisher, Cat# 14-9811-82) is a mouse monoclonal antibody validated by the manufacturer for human samples in flow cytometry applications.<br>Anti-Osteopontin (clone 2F10, Thermo Fisher, Cat# 14-9096-82) is a mouse monoclonal antibody validated by the manufacturer for flow cytometry in human and mouse samples.<br>Anti-MIF (Thermo Fisher, Cat# PA5-27343) is a rabbit polyclonal antibody validated by the manufacturer for use in human tissues in

Western blot and immunohistochemistry applications.
Anti-CD44 (clone IM7, Thermo Fisher, Cat# 14-0441-82) is a rat monoclonal antibody validated by the manufacturer for use in human samples in flow cytometry.
Anti-E-Cadherin (CD324) (clone DECMA-1, Thermo Fisher, Cat# 16-3249-82, RRID: AB_10734213) is a mouse monoclonal antibody validated by the manufacturer for immunofluorescence and for use in human samples. The antibody showed membrane-localized staining consistent with known E-Cadherin expression in epithelial cells. It has been widely used in published studies involving human tissues.
Anti-CD45 (Intracellular Domain) (clone D9M8I, Cell Signaling Technology, Cat# 13917T, RRID: AB_2799583) is a rabbit monoclonal antibody validated by the manufacturer for immunofluorescence in human samples. In our study, it produced specific intracellular staining patterns consistent with known CD45 expression in hematopoietic cells.
Anti-N-Cadherin (clone 5D5, Abcam, Cat# ab98952, RRID: AB_10696943) is a mouse monoclonal antibody validated by the manufacturer for immunofluorescence and for use in human tissues. In our study, the antibody showed strong membrane staining consistent with known N-Cadherin localization in mesenchymal cell populations.
Anti-Keratin 18 (Sigma-Aldrich, Cat# SAB4501665, RRID: AB_10746153) is a rabbit polyclonal antibody validated by the manufacturer for immunofluorescence and for use in human samples. The antibody produced strong cytoplasmic staining consistent with known expression of Keratin 18 in epithelial cell types.
Anti-Tubulin β 3 (TUBB3) (clone TUJ1, BioLegend, Cat# 801213, RRID: AB_2313773) is a mouse monoclonal antibody validated by the manufacturer for immunofluorescence and for use in human samples. This antibody is widely used as a neuronal marker and produced strong cytoplasmic staining in TUBB3+ cell populations consistent with known expression patterns.

Eukaryotic cell lines

Policy information about cell lines and Sex and Gender in Research

Cell line source(s): This iPSC line (WTC-11) generously provided by Dr. Bruce R. Conklin (Gladstone Institute of Cardiovascular Disease, UCSF)

Authentication: Induced pluripotent stem cell line derived from adult skin (leg) fibroblasts; subject clinically normal; wild-type; FISH test: 46, XY. This iPSC line (WTC-11) was produced by Dr. Bruce R. Conklin (Gladstone Institute of Cardiovascular Disease, UCSF)

Mycoplasma contamination: All cell lines were routinely tested every two weeks to ensure that they were not contaminated with Mycoplasma

Commonly misidentified lines (See ICLAC register): No commonly misidentified cell lines listed by the International Cell Line Authentication Committee (ICLAC) were used in this study.

Plants

Seed stocks: *Report on the source of all seed stocks or other plant material used. If applicable, state the seed stock centre and catalogue number. If plant specimens were collected from the field, describe the collection location, date and sampling procedures.*

Novel plant genotypes: *Describe the methods by which all novel plant genotypes were produced. This includes those generated by transgenic approaches, gene editing, chemical/radiation-based mutagenesis and hybridization. For transgenic lines, describe the transformation method, the number of independent lines analyzed and the generation upon which experiments were performed. For gene-edited lines, describe the editor used, the endogenous sequence targeted for editing, the targeting guide RNA sequence (if applicable) and how the editor was applied.*

Authentication: *Describe any authentication procedures for each seed stock used or novel genotype generated. Describe any experiments used to assess the effect of a mutation and, where applicable, how potential secondary effects (e.g. second site T-DNA insertions, mosiacism, off-target gene editing) were examined.*
